# Isolation of primary microglia from the human post-mortem brain: effects of ante- and post-mortem variables

**DOI:** 10.1186/s40478-017-0418-8

**Published:** 2017-02-17

**Authors:** Mark R. Mizee, Suzanne S. M. Miedema, Marlijn van der Poel, Adelia, Karianne G. Schuurman, Miriam E. van Strien, Jeroen Melief, Joost Smolders, Debbie A. Hendrickx, Kirstin M. Heutinck, Jörg Hamann, Inge Huitinga

**Affiliations:** 1 Netherlands Brain Bank, Netherlands Institute for Neuroscience, Amsterdam, The Netherlands; 2 Department of Neuroimmunology, Netherlands Institute for Neuroscience, Amsterdam, The Netherlands; 3 Department of Astrocyte Biology and Neurodegeneration, Netherlands Institute for Neuroscience, Amsterdam, The Netherlands; 4 Department of Experimental Immunology, Academic Medical Center, University of Amsterdam, Amsterdam, The Netherlands

**Keywords:** Post-mortem human brain, Primary human microglia, Rapid cell isolation protocol, Primary microglial cell culture, Biobanking

## Abstract

Microglia are key players in the central nervous system in health and disease. Much pioneering research on microglia function has been carried out in vivo with the use of genetic animal models. However, to fully understand the role of microglia in neurological and psychiatric disorders, it is crucial to study primary human microglia from brain donors. We have developed a rapid procedure for the isolation of pure human microglia from autopsy tissue using density gradient centrifugation followed by CD11b-specific cell selection. The protocol can be completed in 4 h, with an average yield of 450,000 and 145,000 viable cells per gram of white and grey matter tissue respectively. This method allows for the immediate phenotyping of microglia in relation to brain donor clinical variables, and shows the microglia population to be distinguishable from autologous choroid plexus macrophages. This protocol has been applied to samples from over 100 brain donors from the Netherlands Brain Bank, providing a robust dataset to analyze the effects of age, post-mortem delay, brain acidity, and neurological diagnosis on microglia yield and phenotype. Our data show that cerebrospinal fluid pH is positively correlated to microglial cell yield, but donor age and post-mortem delay do not negatively affect viable microglia yield. Analysis of CD45 and CD11b expression showed that changes in microglia phenotype can be attributed to a neurological diagnosis, and are not influenced by variation in ante- and post-mortem parameters. Cryogenic storage of primary microglia was shown to be possible, albeit with variable levels of recovery and effects on phenotype and RNA quality. Microglial gene expression substantially changed due to culture, including the loss of the microglia-specific markers, showing the importance of immediate microglia phenotyping. We conclude that primary microglia can be isolated effectively and rapidly from human post-mortem brain tissue, allowing for the study of the microglial population in light of the neuropathological status of the donor.

**Electronic supplementary material:**

The online version of this article (doi:10.1186/s40478-017-0418-8) contains supplementary material, which is available to authorized users.

## Introduction

Microglia are brain-resident phagocytic cells, which originate from a population of myeloid progenitors from the yolk sac during embryonic development [16, 23, 35] and are maintained through self-renewal without influx of peripheral cells during adult life [1, 4]. Microglia are key players in central nervous system (CNS) homeostasis, fulfilling essential roles in neurodevelopment, adult synaptic plasticity, and brain immunity [32, 34]. In the adult brain, microglia act as surveyors of the local environment to sustain homeostasis and are therefore highly sensitive to changes associated with damage, inflammation, or infection within and outside the CNS. In order to interact with their environment, microglia exhibit a broad range of sensory mechanisms and specific cellular responses, the outcome of which can be both neuroprotective as well as a neurotoxic [22].

During the process of normal aging, the microglial phenotype appears to shift to a primed or more active-prone state [22, 30], the main reasoning behind microglia being linked to pathology in neurodegenerative disorders such as Alzheimer’s disease (AD) [21], Parkinson’s disease (PD) [33], and multiple sclerosis (MS) [24]. Their role as possible contributors to disease has been complemented by evidence for their involvement in the pathophysiology of developmental and psychiatric disorders, such as major depression disorder, bipolar disorder, schizophrenia, and autism [3, 7], either through modulation of neuroinflammation or neuronal plasticity. However, their role in disease pathology appears ambiguous since microglia also display beneficial and restorative functions [36].

Research on microglia function and their role in health and disease has mostly been carried out ex vivo using immunohistochemistry and in vivo using murine models. The isolation of microglia from the brains of various genetic mouse models has greatly facilitated our understanding of basic microglia characteristics in health and disease [9]. Nevertheless, these models are of limited value in relation to human CNS disorders. Studies into human microglia function have highlighted similarities but also crucial differences between mice and humans [38]. Added difficulty comes in the form of various CNS disorders for which animal models are not available or fail to reconstitute important human symptoms. Therefore, to investigate the role of microglia in human context it is crucial to study human primary microglia.

In order to specifically study multiple aspects of human microglia, obtaining pure microglia populations from post-mortem human brain samples is essential. To this aim, we have adapted the human microglia isolation method of Dick et al. [12], in turn based on a rat isolation protocol [37], for the use of post-mortem human brain tissue. This led to a procedure for the rapid isolation of pure human microglia based on cell density separation and capture of CD11b-positive cells using magnetic beads [25]. A major advantage of this isolation procedure in comparison with generally used microglia isolation methods [11] is the omission of effects due to culture and adherence in the procedure, as it allows for direct analysis of isolated microglia. Using this technique, we determined that based on membrane expression of CD45 and CD11b, microglia can be distinguished from autologous peripheral macrophages based on fluorescence intensity [25]. Furthermore, we demonstrated that microglia show a minimal response to lipopolysaccharide (LPS), indicating a tight regulation of inflammatory responses. Finally, we revealed differences in microglial size, granularity, and CD45/CD11b expression in white matter microglia from MS donors, when compared to non-MS donors [26], showing that microglial phenotype reflects neuropathological changes. Yet, to effectively study primary human microglia on a larger scale, there is an urgent need for thorough validation of available protocols and an understanding of the effects of clinical diagnosis and ante- and post-mortem variables on isolated microglia.

Since the development of our procedure for the isolation of human microglia in 2012 [25], we performed microglia isolations from over a hundred brain donors from the Netherlands Brain Bank. In addition to our previously published method, we have also developed a faster protocol that reduces the total isolation time, while maintaining similar or higher viable cell yield. Here we set out to validate the practical aspects of human post-mortem microglia isolations and describe the effects of clinical diagnosis and ante- and post-mortem variables on microglial purity and phenotype, such as post-mortem delay (PMD) and cerebrospinal fluid (CSF) pH, and discuss further application possibilities of isolated human microglia.

## Materials and methods

### Brain tissue

Human brain tissue was obtained through the Netherlands Brain Bank (www.brainbank.nl). The Netherlands Brain Bank received permission to perform autopsies and to use tissue and medical records from the Ethical Committee of the VU University medical center (VUmc, Amsterdam, The Netherlands). On average, the autopsies are performed within 6 h after death. All donors have given informed consent for autopsy and use of their brain tissue for research purposes. The pH of the CSF was measured using a fluid-based pH meter (Hanna Instruments, Nieuwegein, The Netherlands), after rapid sampling of the CSF directly from the lateral ventricles at the start of the autopsy. An overview of the clinical information and post-mortem variables of all brain donors in this study is summarized in Table 1.

**Table 1:** Summary of clinical variables of brain donors used

Diagnosis	Number	Gender (F/M)	Age ± SD	PMD ± SD (hours)	CSF pH ± SD	Total time until processing ± SD
Control	43	1.69	80.91 ± 12.09	6.01 ± 1.31	6.52 ± 0.40	20.01 ± 8.88
AD	17	1.83	80.29 ± 9.92	5.29 ± 0.87	6.35 ± 0.19	19.71 ± 10.75
FTD	6	2	71.50 ± 7.09	5.53 ± 1.62	6.35 ± 0.20	28.44 ± 21.31
MS	32	1.13	65.31 ± 12.15	9.21 ± 1.68	6.46 ± 0.24	20.61 ± 10.01
PD	23	0.53	76.96 ± 10.12	5.77 ± 1.27	6.52 ± 0.24	22.48 ± 8.90
Other	14	0.78	70.25 ± 12.28	6.92 ± 2.77	6.49 ± 0.23	20.33 ± 5.22
All	135	1.17	74.87 ± 12.88	6.71 ± 2.13	6.47 ± 0.30	20.80 ± 10.47

*AD* Alzheimer’s disease, *FTD* fronto-temporal dementia, *MS* multiple sclerosis, *PD* Parkinson’s disease, *OD* other diagnoses (major depression, bipolar disease, neuromyelitis optica, progressive supranuclear palsy), *F* female, *M* male, *SD* standard deviation

### Human post-mortem microglia isolation

**Fig. 1:**
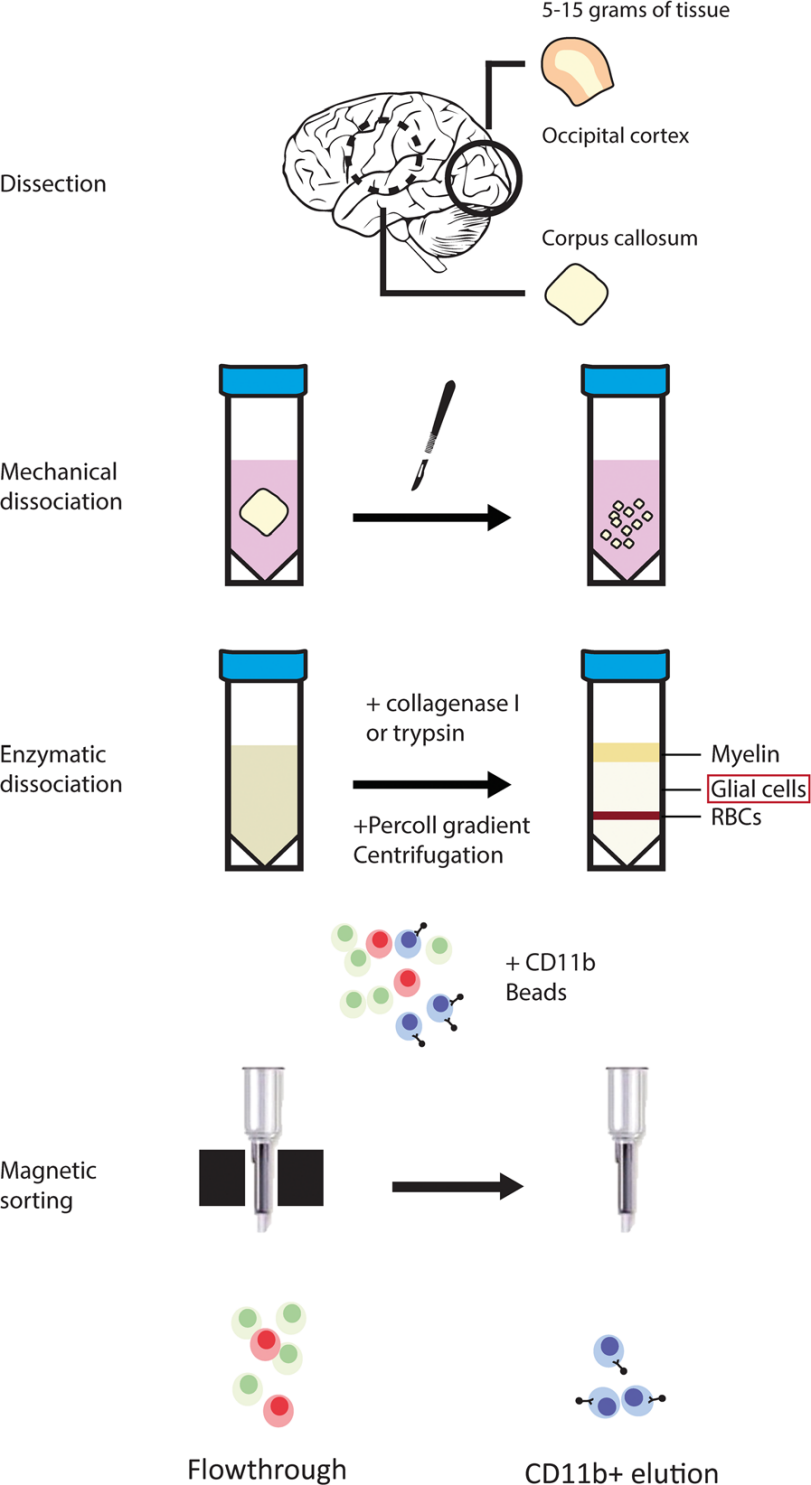
Microglia isolation method at a glance Depicted are the two similar methods through which microglia were isolated from *post*-*mortem* brain tissue. CNS samples were dissected from either occipital cortex (GM), corpus callosum (WM), or subcortical WM. Mechanical disruption of tissue was performed using scalpel (WM) or tissue sieve (GM). Dissociated tissue was then subjected to enzymatic digestion, using DNAse I and either collagenase I (previous method) or trypsin (current method), for 1 h and 45 min respectively. The resulting single cell suspension was subjected to gradient separation using Percoll. The glial cell fraction was extracted, washed, and subjected to CD11b + purification using magnetic beads. CD11b + cells were eluted by removing the column from the magnet and flushing the column

At autopsy, corpus callosum or subcortical white matter (WM) and occipital cortex grey matter (GM) was dissected, collected in Hibernate A medium (Invitrogen, Carlsbad, USA) and stored at 4 °C until processing. Microglia isolations were performed as described previously [25], or through a recently implemented adaptation of this protocol, showing similar or higher yield, while reducing total protocol time to approximately 4 h. The current isolation method and differences with the previous method are depicted, at a glance, in Fig. 1. A point by point, detailed description of the current protocol can be found in the supplemental information. Mechanical dissociation was performed by meshing over a metal tissue sieve, after removal of the meninges (GM) or cutting tissue into fine pieces using a scalpel (WM). Further dissociation was performed by passing the suspension through a 10-ml pipette, followed by enzymatic dissociation with 300 U/ml collagenase 1 (Worthington, Lakewood, USA) for 60’ (previous method) or with trypsin (Invitrogen) at a final concentration of 0.125% for 45’ (current method) in Hibernate A medium at 37 °C on a shaking platform. Both digestions were incubated in the presence of 33 μg/ml DNAseI (Roche, Basel, Switzerland). The digestion was resuspended 10x with a 10-ml halfway the digestion time. Heat inactivated fetal calf serum (FCS, Invitrogen) was added to quench trypsin activity and the cell suspension was centrifuged for 10 min at 1800 rpm and 4 °C. After discarding the supernatant, the cell pellet was resuspended in cold DMEM (Invitrogen), supplemented with 10% FCS, 1% Penicillin-Streptomycin (Pen-Strep, Invitrogen), and 1% gentamycin (Invitrogen), and passed through a 100-μm tissue sieve. After the direct addition of 1/3 volume of cold Percoll (GE Healthcare, Little Chalfont, UK) and centrifugation for 30’ at 4000 rpm and 4 °C the interphase containing microglia was transferred to a new tube (discarding the myelin and erythrocyte layers) and washed two times in DMEM supplemented with 10% FCS, 1% Pen/Strep, 1% gentamycin, and 25 mM Hepes (Invitrogen). Negative selection of granulocytes (previous method only) and positive selection of microglia with respectively anti-CD15 and anti-CD11b conjugated magnetic microbeads (Miltenyi Biotec, Cologne, Germany) was done by magnetic activated cell sorting (MACS) according to the manufacturer’s protocol. Briefly, cells were incubated with 10 μl CD15 microbeads for 15 min at 4 °C, washed, resuspended in beads buffer (0.5% BSA, 2 mM EDTA in PBS pH 7.2) and transferred to an MS column placed in a magnetic holder. The flow-through containing unlabeled cells was collected, washed and subsequently incubated with 20 μl CD11b microbeads for 15 min at 4 °C. Cells were then washed and placed on a new MS column in a magnetic holder. The CD11b^+^ cell fraction was eluted from the column by removing the column from the magnet, adding beads buffer, and emptying the column with a plunger. Viable cells were then counted using a counting chamber and used as described in downstream analyses. The isolation of macrophages was performed using choroid plexus tissue dissected from the lateral ventricle, using the same method as for WM microglia.

### Flow-cytometric analysis

The CD11b + cell fraction was evaluated for proper separation of microglia from other cell types by flow cytometry for CD45 (FITC-labeled, Agilent, Santa Clara, USA), CD11b (PE-labeled, eBioscience, San Diego, USA), and CD15 (APC-labeled, Biolegend, San Diego, USA). For CD45 and CD11b, appropriate isotype controls were regularly included to assess background levels of fluorescence. Cells were incubated with antibodies in beads buffer, on ice, for 30’. Viability of the cells was analyzed using the fixable viability dye Efluor 780 or 7-AAD (eBioscience). For spiking the microglia populations, macrophages where labeled with far red celltracker (Invitrogen) in PBS (1:1000) for 5 min and washed twice with PBS. Fluorescence was measured on either a FACSCalibur or a FACSCanto II machine (both BD biosciences, Franklin Lakes, USA) and analyzed with FlowJo software (Treestar, Ashland, USA). For CD45 and CD11b geometric mean comparisons with post-mortem parameters, only data from the FACS-Calibur was included.

### Cell culture

Microglia were cultured in DMEM/F-12 medium (Invitrogen), supplemented with 10% FCS and 1% Pen-Strep and cultured in plates coated with poly-L-lysine (Invitrogen). Myelin phagocytosis was assessed as described previously [20]. In short, microglia were incubated for 48 h with pHrodo-labeled myelin (10 μg/ml) from a myelin pool containing myelin from 12 donors without neurological abnormalities. All cultures described in the data are derived from white matter samples, as cortical microglia did not result in reproducible cultures. To assess the effect of cryogenic storage and subsequent thawing of primary microglia, cells were resuspended in ice-cold mixture of medium and FCS (1:1), containing 10% dimethyl sulfoxide (DMSO, Sigma, St. Louis, USA), placed in a cryogenic container (Nalgene, Thermo Fischer, Waltham, USA) with 2-propanol, and stored overnight in a −80 °C freezer. Cryovials were then transferred to a liquid nitrogen tank. Cells were thawed by slowly adding cold complete RPMI medium (Invitrogen) containing 20% FCS, after 20 min at room temperature, cells were washed using warm complete RPMI and either lysed for RNA isolation or analyzed directly using flow cytometry.

### RNA isolation and gene expression analysis

Acutely isolated primary microglia were taken up in 1 ml TRIsure (Bioline, London, UK) and stored at −80 °C for further processing. RNA isolation was carried out according to manufacturer’s protocol using phase separation by addition of chloroform and centrifugation, followed by overnight precipitation in isopropanol at −20 °C. RNA concentration was measured using a Nanodrop (ND −1000; NanoDrop Technologies, Rockland, DE, USA) and RNA integrity was assessed using a Bioanalyzer (2100; Agilent Technologies, Palo Alto, CA, USA). cDNA synthesis was performed using the Quantitect reverse transcription kit (Qiagen, Hilden, Germany) according to manufacturer’s instructions, with a minimal input of 200 ng total RNA. Quantitative PCR (qPCR) was performed using the 7300 Real Time PCR system (Applied Biosystems, Foster City, USA) using the equivalent cDNA amount of 1–2 ng total RNA used in cDNA synthesis. SYBRgreen mastermix (Applied Biosystems) and a 2 pmol/ml mix of forward and reverse primer sequences were used for 40 cycles of target gene amplification. An overview of forward and reverse sequences for each gene can be found in Additional file 1: Table S1. Expression of target genes was normalized to the average cycle threshold of GAPDH and EF1a. Cycle threshold values were assessed with SDS software (Applied Biosystems).

### Statistical analysis

Data analysis was performed using Graphpad Prism software (v6 Graphpad Software, La Jolla, CA, USA). Results are shown as mean with standard error of the mean, and statistical analysis was performed using either parametric or non-parametric testing, based on the outcome of the Shapiro-Wilk normality test. The applied test for each calculated value is described in the figure legends.

## Results

### Isolation and characterization of microglia from post-mortem CNS tissue

**Fig. 2:**
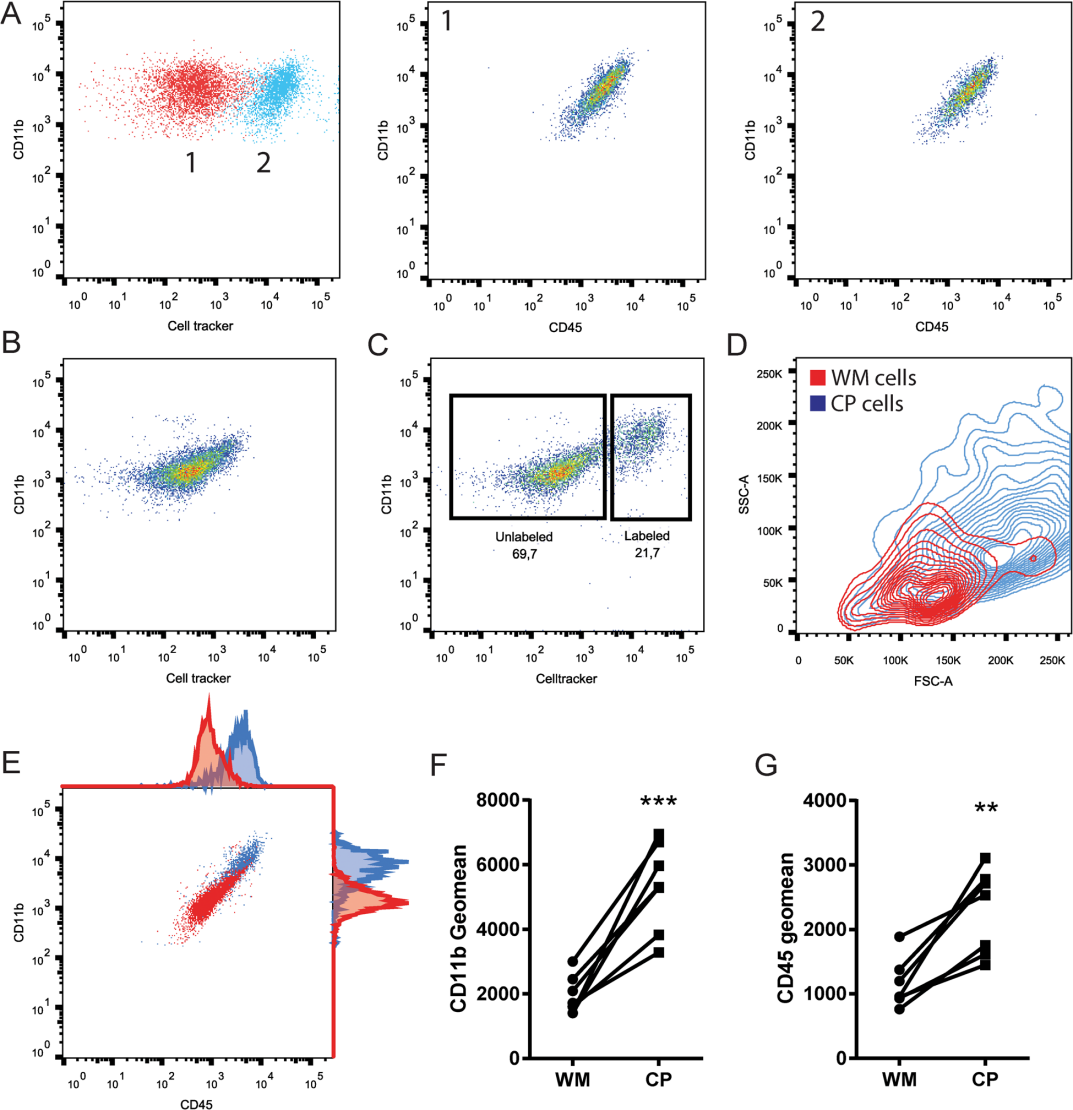
Isolated microglia from post-mortem human CNS tissue are distinguishable from autologous macrophages. **a** FACS plot showing non-labeled (*red*) and far red cell tracker-labeled (*blue*) populations of CP-derived macrophages, CD11b/CD45 expression for both populations are shown in the FACS plot of the corresponding number. **b** FACS plot showing a non-labeled population of WM microglia, note the absence of cell tracker signal. **c** FACS plot showing a mixed population of cell tracker-labeled CP macrophages and non-labeled WM microglia, CP-derived macrophages are clearly separated by cell tracker labeling. **d** Contour plot showing the forward (FSC-A) and sideward (SSC-A) scatter distribution of non-labeled WM microglia (*red*) and cell tracker-labeled CP macrophages (*blue*), showing distinct population size and granularity for each group. **e** The same population of mixed cells as in C, showing CD11b and CD45 immunolabeling, showing increased staining for both markers in CP macrophages (*blue*) compared to WM microglia (*red*). **f**-**g** Quantification of the same cell tracker labeling strategy from seven brain donors shows that CD11b and CD45 geomean is increased in CP macrophages compared to WM microglia for all isolations (paired *t*-test). **p value < 0.01, ***p value < 0.001

The isolation of viable microglia from post-mortem human CNS tissue has been described by our group previously [25]. For the data used in this study, we have used both the published protocol as well as an adapted version that is faster (~4 in place of ~5 h) in which collagenase is replaced by trypsin, and CD15 depletion is omitted. The basic steps of the protocol and the aspects that differ between both protocols are depicted in Fig. 1. The cell capture in both methods relies on the membrane expression of CD11b, which is also present on perivascular and infiltrated macrophages in the CNS. To investigate the differences between macrophages and microglia from the same donor, we included choroid plexus (CP) macrophages. To differentiate between the two populations of cells, CP-derived CD11b^+^ cells were labeled with a fluorescent cell tracker. To ensure that the labeling method did not alter the fluorescence intensity of CD45 and CD11b antibodies, unlabeled and labeled CP macrophages were compared, showing no change in CD45 and CD11b fluorescence (Fig. 2a). Furthermore, we observed no APC/cell tracker^+^ cells in the CD11b^+^ population isolated from WM (Fig. 2b). Representative FACS plots showing the gating strategy to investigate only viable cells, including assessment of background fluorescence using isotype controls, is shown in Additional file 1: Figure S1. Spiking the WM CD11b^+^ cells with labeled CP CD11b^+^ cells enabled us to stain a combined population of WM and CP cells for CD45 and CD11b, while allowing separation of the populations based on APC^+^ (Fig. 2c). Comparing the size and granularity of both cell populations in one pool of cells identified CD11b^+^ cells from WM to have different population characteristics compared to CD11b^+^ cells from CP, showing the macrophages to be larger and more granular (Fig. 2d). Furthermore, CP-derived macrophages clearly showed a higher expression of CD45 and CD11b, when compared to WM-derived cells (Fig. 2e). Quantification of the same analyses from seven different donors with different neurological diagnoses showed that the observations regarding CD45 (avg. 190.8% higher expression levels; Fig. 2f), and CD11b (avg. 106.4% higher expression levels; Fig. 2g) are consistent for all investigated donors. We conclude that microglia can be reliably isolated from post-mortem human CNS tissue, without apparent macrophage contamination due to the fact that a large reservoir of macrophages is not present in the CNS parenchyma.

### Viable microglia yield from white and grey matter correlates with CSF pH

**Fig. 3:**
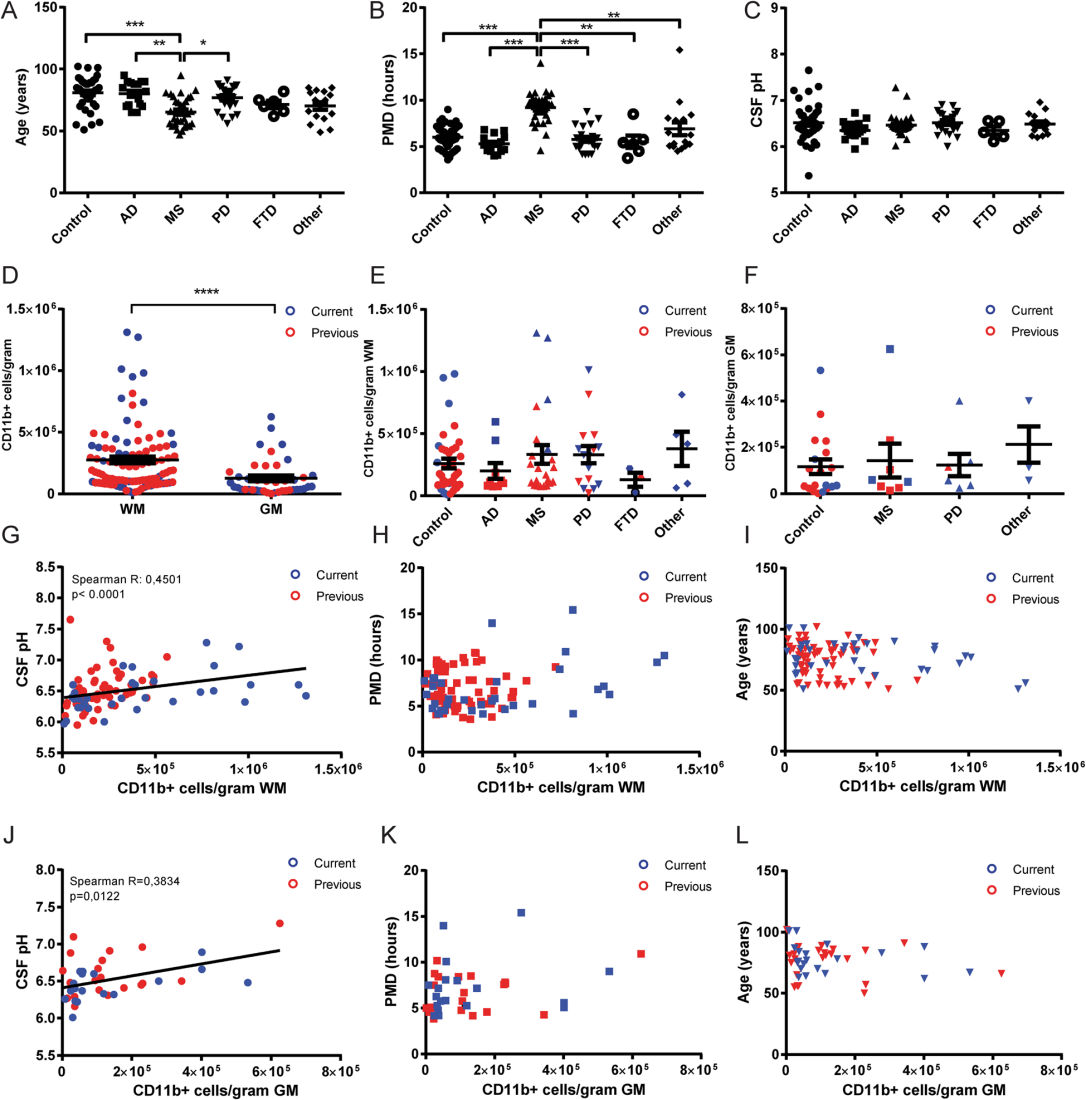
Viable microglia yield is correlated with CSF pH, not age or PMD. **a**-**c** Scatterplots showing the distribution of age, PMD, and CSF pH across donor groups. The MS donor group shows significant differences in both age and PMD compared to other groups (one way ANOVA, Dunn’s multiple comparison test). Note that CSF pH is not related to neurological diagnosis. **d** The number of microglia isolated per gram tissue is higher in WM compared to GM isolations (unpaired Mann-Whitney test). Isolations performed using the previous method are denoted in *red*, those using the current method in *blue*, continued in following graphs. **e**-**f** Microglia yield per gram of WM or GM tissue from different neurological groups shows no differences due to diagnosis (one way ANOVA, Dunn’s multiple comparison test). **g**-**i** Microglia yield from WM tissue shows a significant positive correlation with CSF pH, but not with PMD or age (Spearman correlation). **j**-**l** Microglia yield from GM tissue shows a significant positive correlation with CSF pH, but not with PMD or age (Spearman correlation). *p value <0.05, **p value < 0.01, ***p value < 0.001, ****p value < 0.0001

Since post-mortem microglia isolations were performed on brain samples from varying neurological disease and control donors, we first assessed the differences between the various groups of donors with respect to age, PMD, and CSF pH. Only the MS donor group showed a significant deviation from other groups in age (Fig. 3a) and PMD (Fig. 3b), whereas no significant differences were observed in CSF pH at autopsy between groups. (Fig. 3c). The difference in PMD is explained by the longer autopsy protocol for MS donors in which MRI-guided dissection is needed to separate normal-appearing WM (NAWM) from lesioned areas [10], whereas the difference in age is explained by mortality at a younger age in MS. We then combined data from all isolations, which clearly showed a higher yield of viable microglia per gram WM compared to GM tissue (Fig. 3d). This combined graph also shows the high donor-to-donor variability in microglia yield, in both WM and GM isolations. Colors separating the isolations performed using the two described methods showed that the current trypsin method produced the highest yields, although the average yield between the two methods is not significantly different (Additional file 1: Figure S2).

Since the region-specific difference in microglia yield could be caused by an inherent difference between WM and GM microglia, we separately analyzed isolations from WM and GM to correlate with donor clinical parameters. We first analyzed the influence of a neurological diagnosis on microglia yield. Although both the AD and FTD groups showed lower WM microglia yield averages compared to the control, MS, and PD groups (Fig. 3e), the average number of microglia isolated from WM and GM (Fig. 3f) was not significantly different between groups. We next analyzed the effect of donor age, PMD, and CSF pH on microglia yield. For WM microglia isolations, we observed a significant correlation of viable microglia yield with CSF pH (Fig. 3g), but no correlation with either PMD (Fig. 3h) or age (Fig. 3i). Although the average yield from GM microglia isolations was much lower than those from WM, we observed a similar significant correlation of GM microglia yield with CSF pH (Fig. 3j) and similarly no correlation with either PMD (Fig. 3k) or age (Fig. 3l). Besides investigating PMD, we also included the total time until tissue processing (PMD + time until isolation; averaging 20.8 h over all isolations) in our analysis, which did not show any correlation to microglia yield (Additional file 1: Figure S3).

Combined, our data encompassing microglia isolations from over 100 donors clearly shows a robust effect of CSF pH, shown to reflect cortical pH at autopsy [19], on viable microglia yield from post-mortem brain tissue. We have analyzed the clinical information of all donors to determine which variables correlate with CSF pH. In our donor group, the cause of death, often reflecting the agonal state of the donor before passing, is associated with CSF pH (Additional file 1: Figure S4) and shows that the average CSF pH is significantly lower in donors that suffered from cachexia or pneumonia before death, compared to donors that underwent euthanasia.

### Changes in microglia expression of CD45 and CD11b are mainly attributable to differences between grey and white matter, and neurological diagnosis

**Fig. 4:**
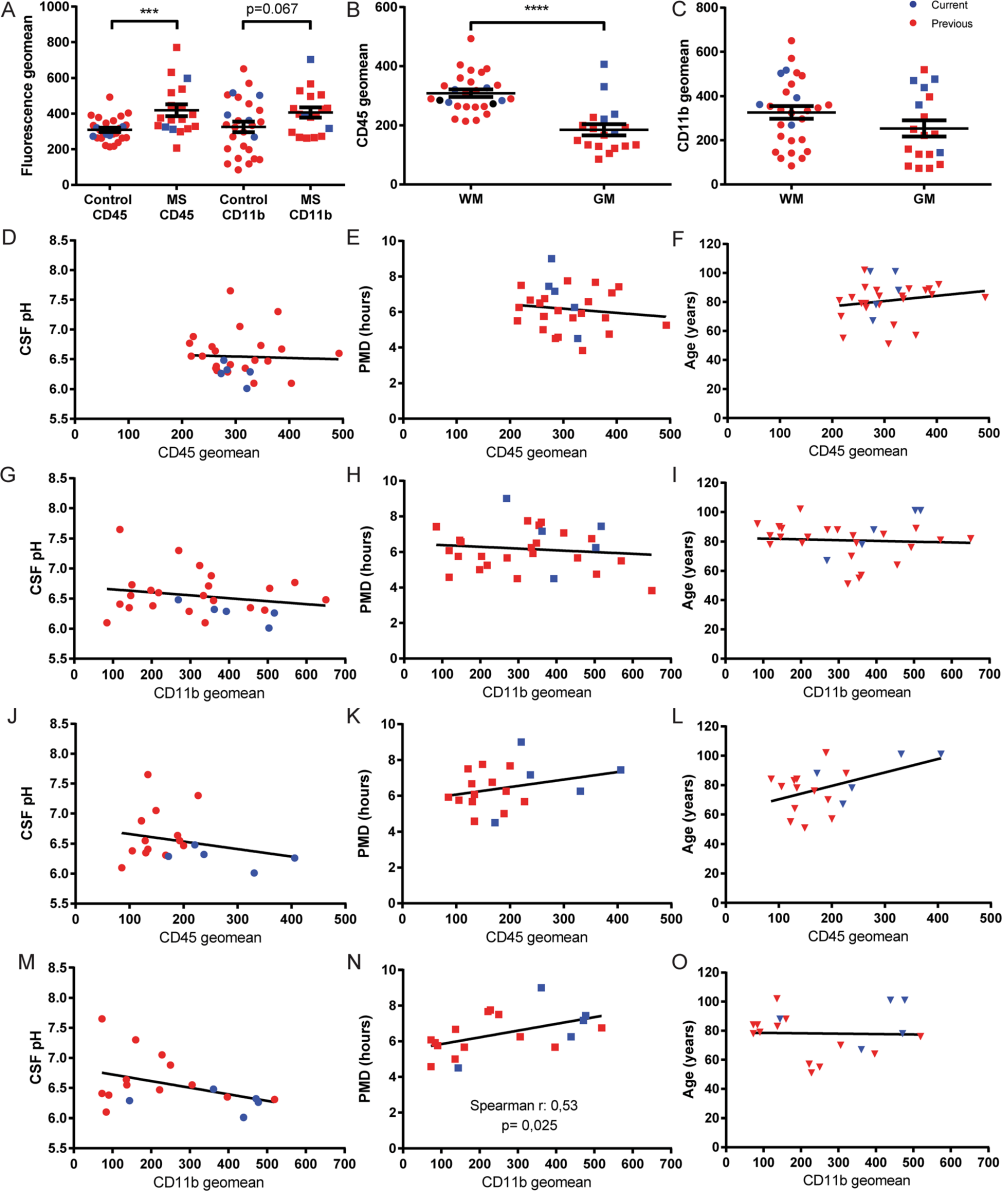
Microglia phenotype in relation to diagnosis and donor variables. **a** Fluorescence geometric means for CD45 and CD11b of microglia isolated from MS or control WM tissue. CD45 expression is significantly higher, CD11b expression does not reach significance (unpaired *t* test). Isolations performed using the previous method are denoted in *red*, those using the current method in *blue*, continued in following graphs. **b**-**c** Fluorescence geometric mean for CD45 and CD11b expression of microglia from WM and GM from non-demented control donors only. CD45 expression but not CD11b expression of WM microglia is increased compared to GM microglia (Mann-Whitney test). **d**-**f** Correlation plots of fluorescence geometric mean of CD45 expression by WM microglia show no significant correlation with CSF pH, PMD, or age (Pearson correlation). **g**-**i** Correlation plots of fluorescence geometric mean of CD11b expression by WM microglia show no significant correlation with CSF pH, PMD, or age (Pearson correlation). **j**-**l** Correlation plots of fluorescence geometric mean of CD45 expression by GM microglia show no significant correlation with CSF pH, PMD, or age (Pearson correlation). **m**-**o** Correlation plots of fluorescence geometric mean of CD11b expression by GM microglia shows a significant positive correlation with PMD, but not with CSF pH or age (Pearson correlation). ***p value < 0.001, ****p value < 0.0001

In order to investigate whether microglia show an altered phenotypical state when isolated from different donor groups, due to varying levels of CSF pH, or under the influence of post-mortem variables like PMD, we performed minimal phenotyping of the isolated microglia. We previously showed increased CD45 expression by microglia derived from MS NAWM compared to non-MS WM [26] as well as by WM microglia isolated from donors with a high degree of peripheral inflammation [25]. Using an extended group of non-demented controls and MS donors, we confirm the elevated CD45 expression in microglia from WM of MS donors (Fig. 4a). CD11b expression was also elevated in microglia from WM of MS donors, but did not reach significance (*p* = 0.067). The same analysis of CD45 and CD11b expression of GM microglia from MS and control donors showed no difference in mean fluorescence (Additional file 1: Figure S5). Therefore, to exclude any effects of disease-related changes in microglia activation, we have only included isolations performed on non-demented control donor material in the following analyses. Using CD45 and CD11b immunoreactivity as a readout for microglial activation state, we analyzed microglia isolated from either WM or GM tissue. Interestingly, we observed a significantly lower membrane expression of CD45 of microglia isolated from GM, when compared to WM-derived microglia (Fig. 4b), whereas CD11b expression is not significantly different (Fig. 4c). Since we also observed a difference in microglia yield from both regions, we separately investigated the effect of clinical and post-mortem parameters on microglia from WM and GM isolations. The membrane expression of CD45 and CD11b of microglia isolated from WM tissue did not correlate significantly with either CSF pH, PMD, or age (Fig. 4d-i). The CD45 expression pattern for microglia isolated from GM was comparable to that of WM microglia, showing no significant correlation with any of the parameters investigated (Fig. 4j-l). In microglia isolated from GM, CD11b expression shows no correlation with CSF pH or age (Fig. 4m, o). Differently from WM microglia however, CD11b expression in GM microglia significantly correlates with increasing PMD (Fig. 4n). We have also included total time until tissue processing in our analysis, showing no correlation with either CD45 or CD11b expression (Additional file 1: Figure S6).

Taken together, our data show that microglial CD45 expression clearly differs between cells isolated from WM or GM. Average CD45 expression on microglia isolated from either WM or GM is unrelated to CSF pH, PMD, age, and population viability. We show a similar absence of correlations for CD11b in both GM and WM microglia, with the only exception being that GM microglia showed increasing CD11b expression with increasing PMD. By combining data of both microglia isolation methods, we also observed a significant increase in both CD45 and CD11b expression of GM microglia isolated using the current method, compared to the previous protocol (Additional file 1: Figure S7). This difference was not observed for WM microglia.

### In vitro applications of primary human microglia and effects of cryogenic storage

**Fig. 5:**
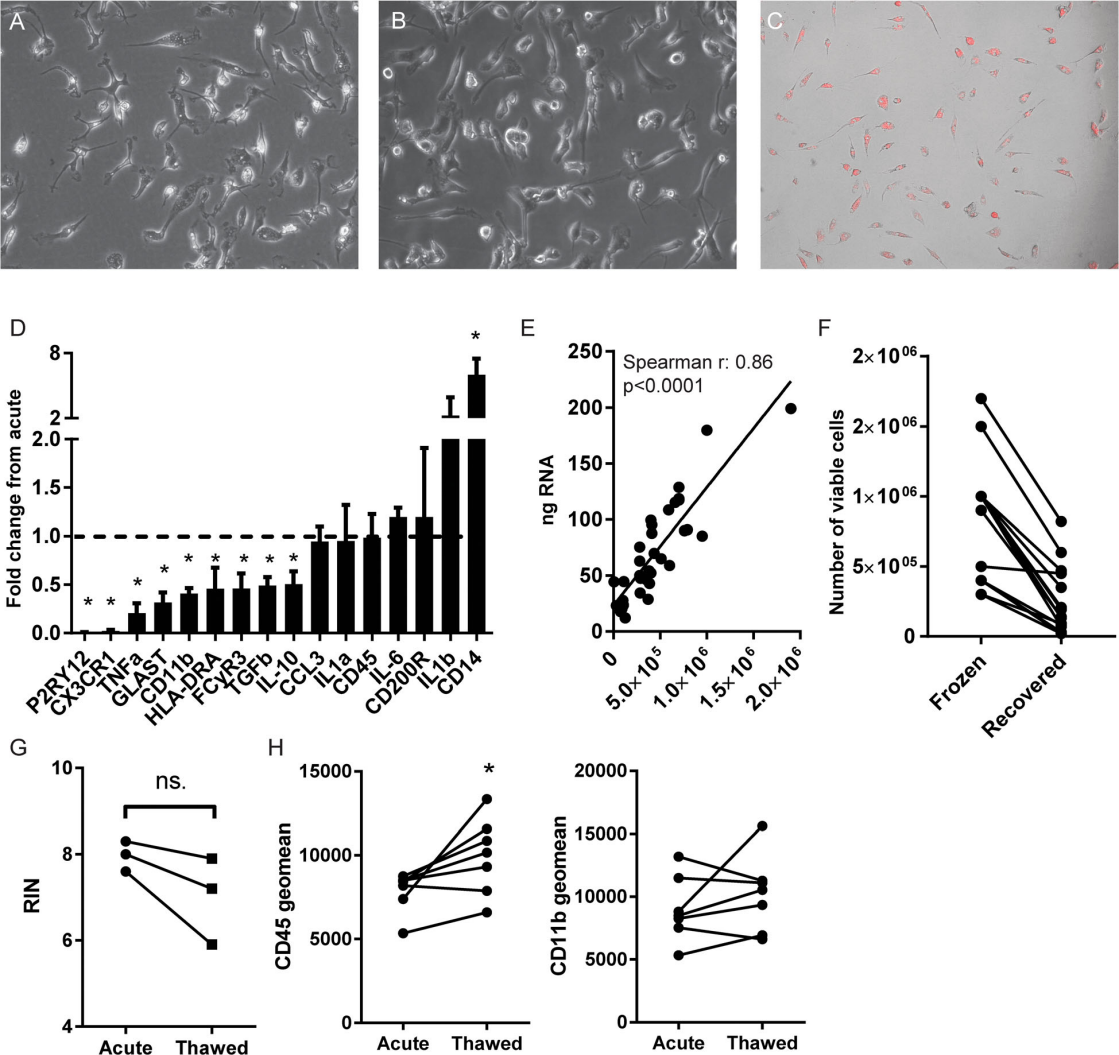
Culture and cryogenic storage of human primary microglia. **a**-**b** Representative phase contrast images of WM microglia under basal culture conditions showing cells with a slightly ramified morphology cultured for 5 days and 10 days respectively (x200). **c** Phase contrast image (x100) of WM microglia incubated with pHrodo-labeled myelin for 48 h at 5 DIV. Superimposed red fluorescence signal shows labeled myelin in phagosomes. **d** Gene expression analysis of microglia after 4 DIV compared to acutely lysed cells, expressed as fold change from acute (Mann-Whitney tests, *n* = 4). **e** Correlation plot of RNA yield with starting number of microglia (Spearman correlation). **f** Linked scatterplot showing the recovery of viable microglia after cryogenic storage. Cells from both WM and GM were used (*n* = 15). **g** RNA integrity of samples from cryogenically stored microglia is not significantly decreased compared to acutely lysed samples (Wilcoxon matched-pairs test). **h** Fluorescence geometric mean of CD45 and CD11b expression of WM microglia before and after cryogenic storage shows that CD45, but not CD11b expression is increased due to freezing (Wilcoxon matched-pairs test). *p value <0.05

To expand the possible research applications of primary human microglia, we investigated the possibility to cryogenically store microglia for biobanking purposes and their potential for (long-term) in vitro culture. Using poly-L-Lysine as a culture substrate, we found that primary microglial cultures show a slightly ramified morphology and can be maintained for 5 days in vitro (DIV) (Fig. 5a) and 10 DIV (Fig. 5b) without apparent signs of proliferation or cell death. Accordingly, immunocytochemistry for proliferation marker Ki-67 only sporadically decorated microglia nuclei (Additional file 1: Figure S8). All microglial cultures were derived from WM samples, as microglia cultures from GM isolations showed no adherence or outgrowth past 2 days in culture. Microglia retain phagocytic function after 5 DIV, as evidenced by the uptake of pHrodo-labeled myelin (Fig. 5c). How the cultured microglial phenotype compares to the phenotype directly after isolation however, has not been addressed to date. We therefore used microglia isolated from four different WM donors, isolated RNA either directly after isolation or after 4 days of basal culture, and investigated the change in gene expression from acute to cultured microglia for each donor (Fig. 5d). Of all investigated genes, only the macrophage marker and lipopolysaccharide co-receptor CD14 was significantly upregulated after 4 days. Interestingly, the microglia/macrophage markers purinergic receptor P2Y12 (P2RY12), fractalkine receptor (CX3CR1), and CD11b were all significantly decreased after 4 days. Moreover, the pro-inflammatory cytokine interleukin 1 beta (IL-1b) showed an increase in expression, but did not reach significance, and immune-activated genes were downregulated, including pro-inflammatory tumor necrosis factor (TNF), glutamate aspartate transporter (GLAST), MHC class II subunit HLA-DRA, Fc gamma receptor IIIa (CD16a), and anti-inflammatory interleukin 10 (IL-10) and transforming growth factor beta (TGFβ). Gene expression of interleukin 1 alpha (IL-1α), chemokine C-C motif chemokine ligand 3 (CCL3), interleukin 6 (IL-6), CD45, and the CD200 receptor (CD200R) was unchanged. Using this selected set of genes, it becomes apparent that microglia undergo phenotypical changes during culture.

Since RNA analysis directly after isolation is important to accurately relate microglial phenotype to the in situ state of the tissue, we analyzed whether RNA yield is constant between donors. We found a significant correlation between the number of viable cells used and the RNA yield obtained (Fig. 5e). Finally, we analyzed the potential to cryogenically store acutely isolated microglia, and the effect of a freeze-thaw cycle on RNA integrity and minimal phenotype. The average recovery rate of viable cells from frozen samples was 27%, although highly variable (±22.7%, Fig. 5f). We analyzed the RNA integrity (RIN) from RNA extracted from microglia immediately after isolation, and after cryogenic storage, from the same donors. Although RIN values were slightly decreased, we found no significant decrease of RIN values after thawing and RIN values did not drop below 6, reflecting usable mRNA in many applications (Fig. 5g). We furthermore analyzed CD45 and CD11b expression on viable microglia before and after thawing. CD11b expression was not significantly affected by cryogenic freezing and thawing (Fig. 5h), but CD45 expression was increased in thawed microglia compared to acutely analyzed cells, possibly reflecting ongoing cell activation or the selective loss of cells with low CD45 expression. Thus, albeit a small sample size, we show that microglia can be cryogenically frozen and stored for biobanking purposes while maintaining the possibility to phenotype using flow cytometry or to analyze gene expression. Furthermore, microglia can be cultured for multiple days, but show profound changes in their gene expression profile due to culture.

## Discussion

In a time-span of 5 years, over a hundred human primary microglia isolations have been performed *on post*-*mortem* human brain samples. Analyzing the results of these efforts, we here confirm that human microglia can be readily isolated from post-mortem CNS tissue based on the membrane expression of CD11b, that microglia are distinguishable from macrophages, and that the yield of viable microglia is linked to the acidification of the CNS at time of autopsy. Strikingly, neither age, PMD, nor neurological diagnosis was correlated with viable microglia yield. The microglia phenotype from control donors, as assessed by CD45 and CD11b expression, was not correlated with brain acidity, donor age, or PMD. We did observe a robust effect of clinical MS diagnosis on CD45 expression, and to a lesser extent on CD11b expression. This finding is of great importance to any study aimed at linking changes in microglial phenotype to a neurological diagnosis. Finally we show that isolated microglia are suitable for culture and cryogenic storage, but provide a cautionary note regarding the changes in microglia gene expression profile due to culture. In summary, the most important conclusion drawn from this study is that after rapid isolation, changes in microglial phenotype can be readily attributable to neurological disease parameters, rather than reflecting uncontrollable donor parameters like age, PMD, idle tissue time, or CNS acidity. This finding is of critical importance to published and future studies implementing the characterization of purified microglia.

The use of purified human microglia to study pathogenic mechanisms of various neurological disorders is relatively new. So far, only a small number of publications exist that describe a microglial phenotype, studying acutely isolated cells with flow cytometry or gene expression analysis, in relation to clinical diagnosis. Our group has previously shown that WM microglia isolated from donors with peripheral inflammation [25] and donors diagnosed with MS [26] display increased size, granularity, and CD45 expression when compared with microglia derived from control donors. Similar findings exist for glioblastoma-derived microglia [29]. These findings clearly demonstrate the potential of purified microglia to shed light on neurological disease processes. There is a growing interest in the use of primary glial cells. A protocol was recently described for the acute purification of human astrocytes from human cortex [40], representing the first description of the molecular profiles for human astrocytes from healthy and tumor tissue, as well as showing a clear distinction between cells from human and mouse origin. Although the advent of genetic animal models resulted in valuable tools to study microglia phenotype and function in animal models of neurological disease [39], the use of human primary cells to study human CNS disorders should gain more traction in the near future. Inevitably, studies that make use of purified human microglia will encounter high inter-donor variation in both cellular yield and experimental read-out. This study, using a relatively large donor sample size is therefore ideally suited to describe donor variables that should be taken into account when analyzing the experimental read-out parameters.

### Microglia yield

Microglia yield is an important factor to address when considering the feasibility of experiments using human microglia. While we did not find any relation of donor age or neurological diagnosis with the cellular yield per gram of brain tissue, CSF acidity at the time of autopsy was a strong indicator of total yield. This finding is in line with previously reported findings on microglia yield using a similar purification method [29]. Interestingly, the same authors reported similar yields from WM, GM, and mixed samples, whereas we observed a clear difference in the number of cells that can be obtained per gram of WM and GM tissue. This finding could reflect a difference in microglia density between WM and GM regions, an observation which has been reported using human post-mortem immunohistochemistry in control brain tissue [28]. Alternatively, a regional difference might exist in the microglial sensitivity to the isolation method. The discrepancy between both studies could be explained by the difference in total sample size. In our donor population, we did not find a correlation between CSF pH and neurological diagnosis or age. As CNS acidity has been shown to relate to agonal state and tissue quality [15], we analyzed our donor population accordingly, and found that the cause of death relates to CSF pH. Donors suffering from cachexia or pneumonia before death showed lower CSF pH than donors that underwent euthanasia. The total microglia yield was not affected by variations in PMD or the total time after death before the tissue was processed. This important finding is in line with previously published findings [29], even though the average PMD of the samples used in the study by Olah et al. in most cases far exceeded our average PMD of 6.7 h. This implies that brain autopsies with long (>12 h) PMDs are still of value to microglia isolations. Combined, this supports the fact that microglia isolations can also be performed by research groups that do not have access to tissue samples within hours after autopsy. Although the average time between death and tissue processing throughout our samples was only 20.8 h, a number of isolations were performed up to 70 h after death, which still yielded viable microglia. Finally, we show that donor age does not influence the microglia yield. Throughout Fig. 3, the combined data are shown for isolations performed using either the previously published or current method. Although the average number of cells/g tissue is higher using the current method, we observed no significant differences between both methods in terms of yield. The currently described method should be preferred however, since it is faster and yields similar or higher microglia numbers.

### Microglia phenotype

Since CD11b does not discriminate between microglia and macrophages and no specific human extracellular microglia marker has been described to date, we wanted to ensure that the CD11b^+^ populations isolated from both WM and GM samples are indeed microglia and are not reflecting the presence of infiltrated macrophages in the parenchyma. Although we have previously shown that macrophages isolated from CP are distinguishable from microglia by size, granularity, and CD45/CD11b expression [25, 26], these analyses were performed on separately isolated populations of cells. To further strengthen the notion that macrophages are not the source of CNS parenchymal CD11b^+^ cells, we developed a way to study both macrophages and microglia in one population. By fluorescently labeling the autologous CD11b^+^ population isolated from CP tissue, and spiking these cells in the parenchymal CD11b^+^ population before minimal phenotyping, we show that microglia and macrophages can be easily distinguished within the same population of cells, by size, granularity and CD45/CD11b expression, similar to findings in murine microglia [22].

The main reason to use an acute and direct purification of microglia from post-mortem CNS samples is to exclude phenotypical changes induced in these cells by prolonged adherence steps used in other isolation protocols [11, 14, 31] as was shown two decades ago by Becher and Antel [2]. Any phenotypical change detected in acutely isolated populations should therefore be relevant to the neuropathological status or CNS location of the samples from which the cells were extracted. We observed a significant difference in CD45 expression, but not CD11b expression when comparing WM and GM microglia from control donors. This finding is in line with the notion of region-specific microglia phenotypes described recently [13, 18] as well as a recent study showing different expression profiles for human microglia from cortex and WM [27]. We show that microglia isolated from MS WM can be distinguished from microglia from control donors based on CD45 expression, reflecting an alerted state [26], as human microglia are known to increase the expression of specific CD45 isoforms upon immune activation [8]. However, the MS donor group, due to disease characteristics and autopsy protocol respectively, also significantly deviates from the control group in age and PMD. It is therefore crucial to be aware of any effect of clinical parameters (other than neurological) on microglia phenotype. Our data clearly show that none of the parameters investigated (PMD, donor age, CSF pH, total time until isolation, and cell viability) had a significant effect on the minimal phenotype. The only exception to these observations was the CD11b expression of GM microglia, for which a positive correlation with PMD was found. These findings strengthen the notion that microglial changes found in acutely isolated populations can be reliably attributed to the neuropathological status of the CNS sample. That being said, clinical parameters in donor groups should be carefully considered, especially for GM microglia comparisons. Furthermore, care should be taken when comparing microglia phenotypes between studies using different isolation methods. We made use of two similar methods where the main difference is the use of either trypsin or collagenase I, both of which are widely used for tissue digestion. Although no differences were apparent in WM microglia phenotype, GM microglia appear to be more sensitive to the choice of method, showing increased CD45 and CD11b immunoreactivity with the current method. Although our sample size for this comparison was small, this could reflect a differential sensitivity of differentiating markers to enzymatic cleavage in WM and GM microglia.

### In vitro culture and cryogenic storage of primary microglia

The immediate analysis of the proteome or transcriptome of acutely isolated microglia will continue to be the most accurate reflection of microglial phenotype in situ. However, functional assays using primary human microglia could provide a unique tool to study functional microglial responses to various stimuli in vitro, either related to neurological disease mechanisms or therapeutic interventions. To understand the outcomes of these types of study, it is crucial to map microglial changes after isolation and during various culturing methods. Our group has previously shown the potential for short-term culture of primary human microglia up to 72 h, in which microglia retain functional properties as evidenced by myelin phagocytosis [20] and response to inflammatory stimuli [25, 27]. We here show that primary WM microglia, in contrast to GM microglia, can be maintained in culture for up to 10 days without overt cell death, and that functional phagocytosis of human myelin can still be induced after 5 culture days. The difference in culturing potential between WM and GM microglia was a surprising finding, as culturing protocols for human GM microglia have been described [14, 31], although mainly for CNS surgery obtained samples. This might reflect a critical time window post-mortem for GM samples after which the microenvironment no longer permits microglia to adhere in culture. Prolonged, or even very short-term culture will have an effect on microglia behavior. This was addressed recently using murine microglia [5, 6], showing the dependence of primary microglia on TGF-beta stimulation to maintain a resting microglia expression profile. Using a select set of genes, we were able to identify major changes in the microglial gene expression due to 4 days of basal culture using primary microglia from multiple brain donors. Although no evident pro- or anti-inflammatory profile could be distinguished, it was clear that the widely used microglia markers P2RY12 [5] and CX3CR1 [17] were decreased in expression level. In contrast, the lipopolysaccharide co-receptor CD14 was highly upregulated, as we and others have shown before with increasing culture time [2, 25]. Our data show that cultured human microglia can be readily used in functional experiments, but it should be stressed that cultured microglia can no longer be compared to their acutely analyzed counterparts. Whether specific culture conditions like the addition of TGFβ1 are also able to skew cultured human microglia to a more resting-like phenotype warrants further investigation. An alternative isolation method to obtain pure populations of primary human microglia from autopsy tissue was recently described, relying on the adherent properties of microglia [31]. When purifying microglia for functional experiments, total cell yield becomes especially important, so the choice of isolation method must be determined by the downstream application. Finally, we explored the possibility of cryogenically storing primary microglia for biobanking purposes. This would allow researchers without direct access to unfixed brain autopsy samples to work with primary human microglia, enhancing the amount of possible scientific investigation. We here show that although cell recovery after cryogenic storage is variable, high quality RNA can still be extracted after cryogenic storage, and minimal cellular phenotyping is still possible. Since we do show effects of cryogenic storage on CD45 expression, acutely analyzed microglia and cryogenically stored microglia should not be compared directly.

## Conclusions

In summary, we show that changes in microglial phenotype, analyzed in an extensive collection of acutely isolated microglia, can be attributed to neurological diagnosis rather than reflect normal variation in ante- and post-mortem parameters. This finding is of critical importance to published and future studies revolving around the characterization of acute cell isolations from human neurological specimens.

## Additional file

Additional file 1: Additional file one contains all supplemental information mentioned in this manuscript: supplemental table 1, supplemental figures 1-8, and the detailed microglia isolation protocol description. (PDF 320 kb)
