# Supplementary material for: Isolation of primary microglia from the human post-mortem brain: effects of ante- and post-mortem variables
Source: Acta Neuropathol Commun. 2017 Feb 17;5:16. doi: 10.1186/s40478-017-0418-8 (PMC5316206; doi:10.1186/s40478-017-0418-8)
Supplement: Additional file 1: — Additional file one contains all supplemental information mentioned in this manuscript: supplemental table 1, supplemental figures 1-8, and the detailed microglia isolation protocol description. (PDF 320 kb) [file 40478_2017_418_MOESM1_ESM.pdf]

Supplemental material

Supplemental table 1. Forward and reverse primer sequences used in this study.

**Primers**	**Forward**	**Reverse**
IL10	TGCCTTCAGCAGAGTGAAGACTT	TCCTCCAGCAAGGACTCCTTTA
IL6	CAGCCACTCACCTCTTCAGAA	TGCCTCTTTGCTGCTTTCACA
CCL18	CCCAGCTCACTCTGACCACT	GTGGAATCTGCCAGGAGGTA
CD200R	GAGCAATGGCACAGTGACTGTT	GTGGCAGGTCACGGTAGACA
CD163	AAGACGCTGCAGTGAATTGCA	GGATCCCGACTGCAATAAAGGAT
HLA-DRA	CCCAGGGAAGACCACCTTT	CACCCTGCAGTCGTAAACGT
FCyr3	AGAATGGCAAAGGCAGGAAGT	AAAAGCCCCCTGCAGAAGTAG
CX3CR1	TTGGCCTGGTGGGAAATTTGT	AGGAGGTAAATGTCGGTGACACT
P2RY12	TGGATACATTCAAACCCTCCAGA	GGTGCACAGACTGGTGTTAC
GLAST	GCTGTGGTGATTGGCATAATCA	CAGCTGTCACTCGTACAATTTTGC
TGFb1	CGCGTGCTAATGGTGGAAA	CTCGGAGCTCTGATGTGTTGAA
IL1b	CCAGCTACGAATCTCGGACCACC	TTAGGAAGACACAAATTGCATGGTGAAGTCAGT
IL1a	CATCGCCAATGACTCAGAGAAG	TGCCAAGCACACCCAGTAGTCTTGCTT
CD14	ACAGGGCGTTCTTGGCTCGC	CGGGAAGGCGCGAACCTGTT
CD11B	TGCTTCCTGTTTGGATCCAACCTA	AGAAGGCAATGTCACTATCCTCTTGA
CD45	GCAGCTAGCAAGTGGTTTGTTC	AAACAGCATGCGTCCTTTCTC
CCL3	GCAGCAGACAGTGGTCAGT	GTGCAGAGGAGGACAGCAA
TNFa	GGCGTGGAGCTGAGAGATA	CAGCCTTGGCCCTTGAAGA
GAPDH	TGCACCACCAACTGCTTAGC	GGCATGGACTGTGGTCATGA
EF1a	AAGCTGGAAGATGGCCCTAAA	AAGCGACCCAAAGGTGGAT

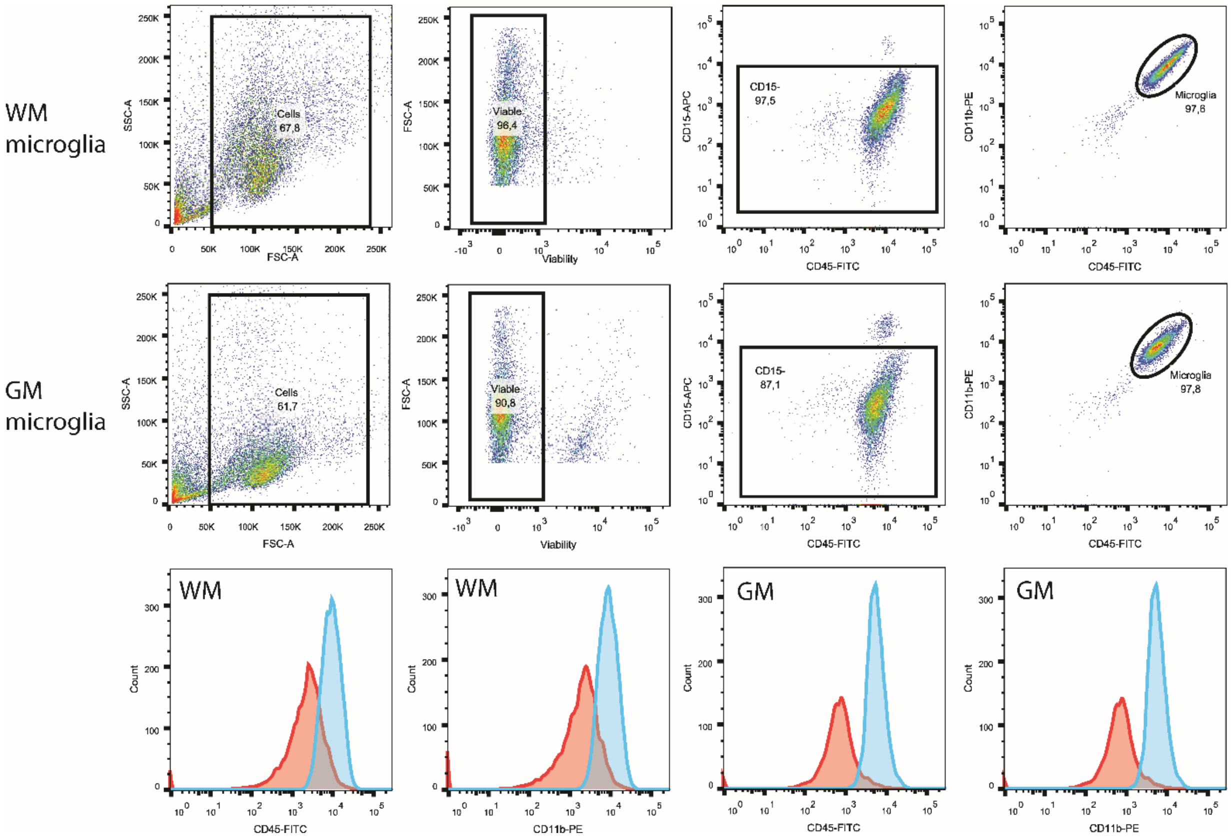

Supplemental figure 1. Representative FACS plots showing the gating strategy used to assess isolation efficiency and microglial surface marker expression. Consecutive steps from left to right: Size and granularity gating to filter out debris, gating on viability dye exclusion, gating on the CD45+/CD15- population, and finally gating for the CD45+/CD11b+ population. The histograms show the appropriate isotype controls (in red) compared to the specific antibodies (in blue).

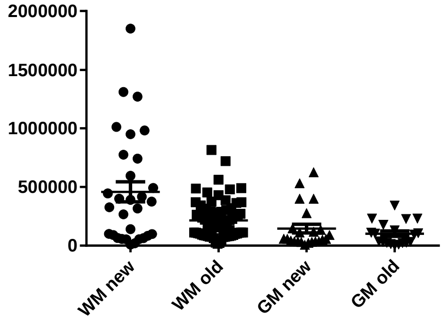

Supplemental figure 2. Viable microglia yield shows no overall significant difference between both isolation methods, in both WM and GM isolations (unpaired Mann-Whitney test).

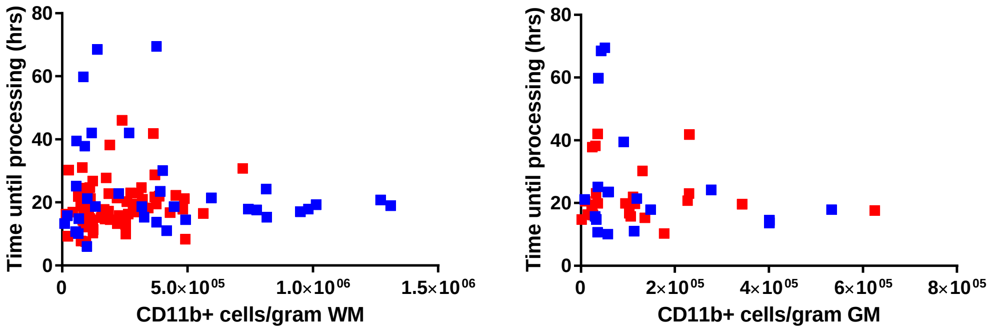

Supplemental figure 3. Scatter plots showing the viable microglia yield and total time until processing. No correlation was found for WM nor for GM isolations.

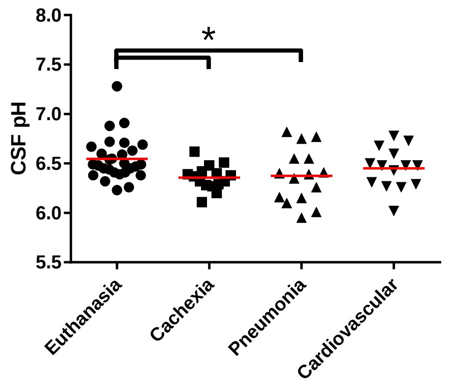

Supplemental figure 4. CSF pH of brain donors shows a correlation with cause of death. Donors that underwent euthanasia showed significantly higher CSF pH than donors that underwent cachexia or pneumonia (one-way ANOVA, Newman-Keuls multiple comparison, * p<0.05)

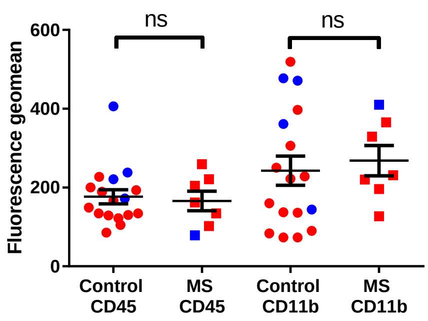

Supplemental figure 5. Fluorescence geometric means for CD45 and CD11b of microglia isolated from MS or control GM tissue.

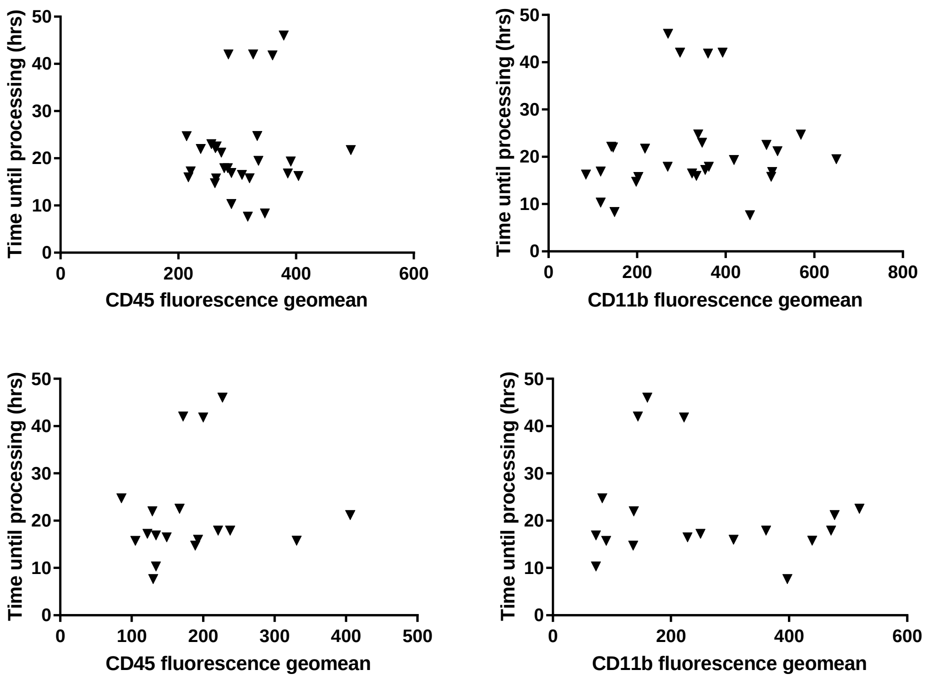

Supplemental figure 6. Fluorescence geometric means of microglia plotted against total time between death and tissue processing for CD45 and CD11b from WM samples (upper panels) and GM samples (lower panel) derived from only non-demented control donors.

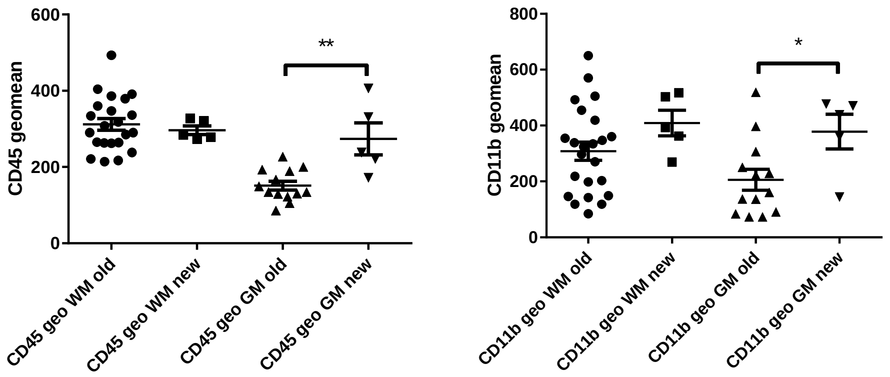

Supplemental figure 7. Microglial CD45 and CD11b geometric means show a select sensitivity of GM microglia to the isolation method, where both CD45 and CD11b expression are higher in cells isolated through the current method (Mann Whitney test, *p<0.05, **p<0,01)

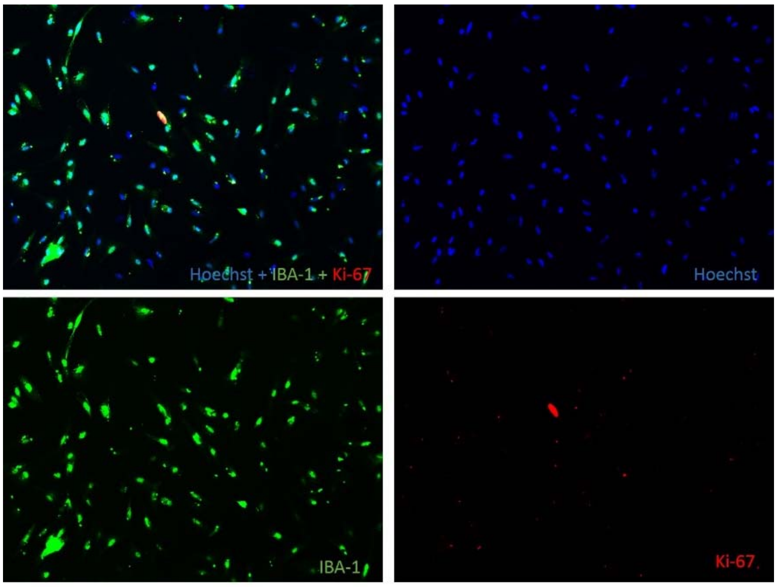

Supplemental figure 8. Representative immunocytochemistry image for ki-67 (red) shows an incidental Iba-1+ (green) microglial DAPI+ blue) nucleus. Microglia were fixed after 4 days in vitro (200x magnification).

Detailed protocol for the isolation of primary human microglia

Tissue

- Collected at autopsy and stored in 50 ml tube containing 25 ml hibernate-A medium at 4°C. Tissue pieces over 5 grams should be cut into smaller sections to maintain enough surface to volume ratio.

Media

- Complete medium (**c**DMEM)
  - 450 ml DMEM (high glucose, with phenol, Invitrogen)
  - 50 ml FBS (10%)
  - 5 ml Pen/Strep (1%)
  - 12.5 ml 1M Hepes buffer (for PH)
  - Optional 0.5 ml gentamycin
- Complete deficient DMEM (**d**DMEM)
  - 450 ml DMEM (without phenol)
  - 50 ml FBS (10%)
  - 5 ml Pen/Strep (1%)
  - Optional 0.5 ml gentamycin

Reagents/buffers

- Clean (autoclaved) Erlenmeyer, 100 mesh filter & cup, scalpels and tweezers
- Hibernate-A (life technologies)
- Trypsin, 2.5% stock (life technologies)
- FBS (life technologies)
- DNAse I 10 mg/ml in braun water (Roche)
- Percoll (GE Healthcare)
- Beadsbuffer: PBS pH 7.2, 0.5% BSA, 2 mM EDTA
- CD11b microbeads, MS columns and magnet setup (Miltenyi)

Protocol

- Weigh tube and calculate amount of tissue. The volumes described below are calculated for use with ~4 grams of tissue. Scale the used reagents used down or up with 2 gram increments (each 2 grams add 15 ml hibernate, 0.75 ml trypsin, and 50 ul DNAse1)
- Put tissue in 10 cm petri dish with hibernate, remove large vessels/membranes. After this step pieces should be pipettable with 10 ml pipette.
  - WM: chop tissue in little chunks using 2 scalpels.
  - GM: grind the tissue over a 100 mesh tissue sieve with a plunger from a 50ml disposable syringe. Flush out any attached tissue from the sieve with Hibernate.

- Pipet tissue-chunks with hibernate in Erlenmeyer, add more hibernate if needed, make sure the final volume is 30ml.
- Add 1.5 ml trypsin stock and 100 ul DNAse1 to 30 ml tissue/hibernate. Seal Erlenmeyer and incubate in shaking incubator (37°C) for 45 minutes.
- Resuspend tissue dissociate ~10x with 10 ml pipette after ~20 minutes, place back in shaking incubator.
- Add 2 ml FBS (at least much ml as trypsin added), resuspend/mix well and put suspension in 50 ml tube. If working with more than 4 grams of tissue, split sample in as many tubes as necessary (max ~4 gr per Percoll tube). Spin sample 10'@1800 rpm 4°C (do not fast cool the centrifuge, so cooling starts when sample is being centrifuged).
- Pour off supernatant and resuspend pellet in ~15 ml cold dDMEM. Filter cell suspension through a clean 100 mesh filter/cup in a petri dish. Add dDMEM if needed (for rinsing petri dish). Collect cells (and "dish-rinse") in a 50 ml tube. Final volume should be 20 ml, also when working with smaller tissue quantities.
- Slowly drip cold Percoll directly on the cell suspension. Use half the volume in tube (eg 20 ml cell suspension: add 10 ml Percoll). Do not mix! Spin tube(s) 30'@4000 rpm 4°C, acc 9, brake 4. After this step carefully remove the tubes from the centrifuge. You should see 3 layers: myelin on top, erythrocytes almost in the bottom, Percoll layer on the bottom. Glial cells are between myelin and erythrocytes.
- Using a glass pipette, transfer cells to a new 50 ml tube (either remove the myelin layer with a pipette or carefully push aside), try not to incorporate any erythrocytes/myelin. Wash cells by filling up tube with cDMEM, spin 10'@1500 rpm 4°C.
- Pour off supernatant and resuspend the pellet in 25 ml cDMEM, spin samples 10'@1200 rpm 4°C.
- Pour off supernatant and resuspend cells in beads buffer when continuing with bead isolation. Count the number of viable cells (cell chamber) using Trypan Blue.

Beads isolation (Miltenyi protocol)

- For beads isolation use cold "beads buffer" (BB, according to protocol). All centrifuge steps are 5'@1220 rpm 4°C (or 300G). Add BB to cells (~3 ml/sample), spin, resuspend cells in 80 µl BB and add 20 µl CD11b beads. Incubate 15 min in fridge, add 5 ml BB, spin and resuspend cells in 500 µl BB. When input exceeds $10 \cdot 10^6$ cells add more beads according to Miltenyi protocol.
- During incubation set up magnet and MS columns

- Wash MS column with 500 µl BB, when reservoir is empty put cells on column. Wash column 3 times by adding 500 µl BB whenever column reservoir is empty. Collect flowthrough and wash steps in one tube (this contains all CD11b-negative cells).
- Elute column: take column off magnet, add at least 1 ml BB and push cells out using plunger immediately after addition of BB.
- For fractions of interest: add buffer/medium to your cells, spin, resuspend and count cell number in a cell chamber using trypan blue. Always reserve cells from cd11b elution (10.000 – 20.000 cells) for staining (CD45, CD11b, CD15) and analysis on FACS.
- Stain cells for FACS analysis (or sorting):
    - 2.5 µl CD11b-PE or 2.5 µl isotype-PE
    - 8 µl CD45-FITC or 8 µl isotype-FITC
    - 3 µl CD15-APC or 3 µl isotype-PE
    - 8 µl human pool serum

  Endvolume = 100 µl per sample, incubate all stainings for 30’ on ice
- Wash cells (fill well with buffer, spin, pour off supernatant) 2 times before FACS analysis.
